# Impact of long-term high-flow nasal therapy on mucus plugs in patients with bronchiectasis

**DOI:** 10.1183/23120541.00962-2024

**Published:** 2025-06-09

**Authors:** Claudia Crimi, Santi Nolasco, Raffaele Campisi, Mattia Nigro, Pietro Impellizzeri, Andrea Cortegiani, Alberto Noto, Andrea Gramegna, Carlo Vancheri, Francesco Blasi, Nunzio Crimi, Stefano Aliberti, Annalisa Carlucci

**Affiliations:** 1 Department of Clinical and Experimental Medicine, University of Catania, Catania, Italy; 2 Respiratory Medicine Unit, Policlinico “G. Rodolico – San Marco” University Hospital, Catania, Italy; 3 Department of Biomedical Sciences, Humanitas University, Milan, Italy; 4 IRCCS Humanitas Research Hospital, Milan, Italy; 5 Department of Precision Medicine in Medical, Surgical and Critical Care (Me.Pre.C.C.), University of Palermo, Palermo, Italy; 6 Department of Anesthesia, Intensive Care and Emergency, Policlinico Paolo Giaccone, University of Palermo, Palermo, Italy; 7 Department of Human Pathology of the Adult and Evolutive Age “Gaetano Barresi”, Division of Anesthesia and Intensive Care, University of Messina, Policlinico “G. Martino”, Messina, Italy; 8 Department of Pathophysiology and Transplantation, Università degli Studi di Milano, Milan, Italy; 9 Internal Medicine Department, Respiratory Unit and Adult Cystic Fibrosis Center, Fondazione IRCCS Cà Granda Ospedale Maggiore Policlinico Milan, Milan, Italy; 10 Respiratory Unit, IRCCS Humanitas Research Hospital, Milan, Italy; 11 Department of Medicina e Chirurgia, Università Insubria, Varese, Italy; 12 Pulmonary Rehabilitation Unit, Istituti Clinici Scientifici Maugeri, Pavia, Italy

## Abstract

Bronchiectasis is a chronic disease characterised by abnormal dilatation of the bronchi and impaired mucus clearance [1]. From a pathophysiological point of view, the evidence suggests that an initial insult triggers a significant inflammatory response, leading to unresolved inflammation, mucus hypersecretion and further mucus obstruction, resulting in progressive and irreversible lung damage and dilatation. This creates a conducive environment for bacterial infections, promoting an inflammatory vicious cycle [2]. As a result, patients often experience chronic cough, sputum production and frequent exacerbations, which significantly jeopardise their quality of life [3].

## Shareable abstract

**Patients with bronchiectasis treated with long-term high-flow nasal therapy showed a significant improvement in mucus plug score** https://bit.ly/3NV39zI

*To the Editor:*

Bronchiectasis is a chronic disease characterised by abnormal dilatation of the bronchi and impaired mucus clearance [1]. From a pathophysiological point of view, the evidence suggests that an initial insult triggers a significant inflammatory response, leading to unresolved inflammation, mucus hypersecretion and further mucus obstruction, resulting in progressive and irreversible lung damage and dilatation. This creates a conducive environment for bacterial infections, promoting an inflammatory vicious cycle [2]. As a result, patients often experience chronic cough, sputum production and frequent exacerbations, which significantly jeopardise their quality of life [3].

Mucus in the airways of individuals with bronchiectasis tends to be dehydrated and more viscous [4], leading to mucus stasis and adhesion to the airway surface that, coupled with mucus hypersecretion, leads to the formation of mucus plugs, particularly in distal airways [5]. Notably, mucus plugs have been identified as a feature linked to airflow obstruction [6], a higher rate of exacerbations [7] and increased mortality in COPD [8], making them a potential biomarker of severity in muco-obstructive lung diseases. Recently, a radiographic mucus score [9, 10] was described to quantify airway mucus plugging using computer tomography (CT) imaging in patients with asthma and COPD. However, quantitative assessment of mucus impaction in the airways of patients with bronchiectasis is rarely considered in daily clinical practice.

Recent studies have shown that high-flow nasal therapy (HFNT) has significant benefits for bronchiectasis patients [11–13]. In a matched case-control study [14], we compared outpatients with bronchiectasis on long-term HFNT to those on optimised medical treatment alone finding a significant reduction in the annual exacerbation rate compared to standard care. In this post-hoc analysis of our case-control study [14], we examined the effect of HFNT on airway mucus plugs evaluated by using chest CT scan in patients with severe bronchiectasis, providing mechanistic insight into the clinical effects of this treatment.

The full methods of the study are described in the original published manuscript [14]. In brief, patients were enrolled if they met all the following criteria: 1) clinically relevant and radiologically confirmed bronchiectasis on chest high-resolution CT [1]; 2) at least one severe exacerbation (defined as an exacerbation requiring hospital admission) in the previous year; 3) optimised medical maintenance therapy, respiratory physiotherapy and pulmonary rehabilitation per European Respiratory Society guidelines [3]. Patients with cystic fibrosis or traction bronchiectasis were excluded. Patients with COPD were included if bronchiectasis was the primary diagnosis. The clinical severity of bronchiectasis was evaluated according to the bronchiectasis severity index (BSI) [14]. We considered the presence of chronic colonisation if the same pathogen was found in at least two sputum cultures with a minimum of 3 months apart for 1 year in stable clinical condition. Only patients who underwent CT scan at baseline and after 12 months were included. The CT scans were performed in volumetric mode with maximal inspiration with a slice thickness of 1–1.25 mm and no interval gap during clinical stability with no history of exacerbation within 4 weeks prior. The CT mucus score was determined using a bronchopulmonary segment scoring system (range 0–20) proposed by Dunican *et al.* [9]. The mucus score was evaluated by two independent pulmonologists (S. Nolasco and R. Campisi) with experience in lung imaging, blinded to clinical information. Any discrepancies were resolved at a consensus meeting [10]. The intraclass correlation coefficient for between-rater mucus score agreement was 0.88 (95% CI 0.74 to 0.96).

HFNT was initiated using a dedicated device (myAirvo 2, Fisher and Paykel Healthcare, Auckland, New Zealand) set at a flow rate of 25–40 L·min^−1^, and at a temperature of 34–37°C, according to patient tolerance. Patients were instructed to use the device for six or more hours per day, preferably at night. The research protocol was approved by the “Catania 1” Ethics Committee of the Policlinico University Hospital (Approval Number 176/2018/PO, Catania, Italy) and adhered to the Declaration of Helsinki.

Categorical variables are stated as numbers (n) and percentages (%). Continuous variables are expressed as the median and interquartile range (IQR). Median differences and 95% confidence intervals (95% CI) were assessed to evaluate treatment effects. Differences between groups were assessed using Wilcoxon rank-sum or Mann–Whitney tests. Linear regression analysis was developed to evaluate the association between BSI and mucus plug score. All statistical tests were two-tailed, and p-values <0.05 were considered statistically significant. Statistical analyses and figures were generated using GraphPad Prism (version 10.1.0) (GraphPad Software, San Diego, CA, USA).

Patients already receiving optimised medical treatment and home HFNT (n=10) were matched 1:1 with a control group of patients (n=10) on optimised medical treatment alone. Matching criteria included age, gender, BSI, exacerbations (number per year) and *Pseudomonas aeruginosa* colonisation.

Patients’ baseline characteristics are described in the original published manuscript [14]. All patients had severe bronchiectasis according to the BSI (median 13 (IQR 11.5–15.5) for the HFNT group and 11 (IQR 10–13.5) for the control group). The median HFNT flow rate was 33 L·min^−1^ (IQR 25–40), with a median temperature of 34°C (IQR 34–37). The HFNT treatment was well tolerated. The median daily duration of HFNT use was 6.5 h per day (IQR 5.8–7.7).

At baseline, the median mucus score was 5.5 (IQR 3.8–7.5) in the HFNT group and 5.5 (IQR 4–6.8) in the control group. In both cohorts, 60% of patients had high mucus scores (≥5). There was no correlation between baseline BSI and mucus score (R=0.007, p=0.7824). Figure 1 summarises the effect of HFNT compared to standard treatment alone on mucus score assessed by CT scan. A reduction in mucus score was found in the HFNT group after 12 months (−2 (95% CI −5 to −0), p=0.0156), with a significant difference of −2 (95% CI −4 to −0), p=0.0221 compared to the control group (figure 1a). Furthermore, the number of lung lobes with mucus plugs was reduced by −1 (95% CI −2 to −0) in the HFNT group *versus* controls (p=0.0061) (figure 1b). Figure 1c shows representative CT slices from two different HFNT group patients taken before and after treatment. Yellow arrows identify mucus plugs at baseline that were resolved after 12 months of HFNT. The percentage of patients with high mucus score remained unchanged in the control group (figure 1d), while it decreased by 20% in the HFNT group (figure 1e).

**FIGURE 1:**
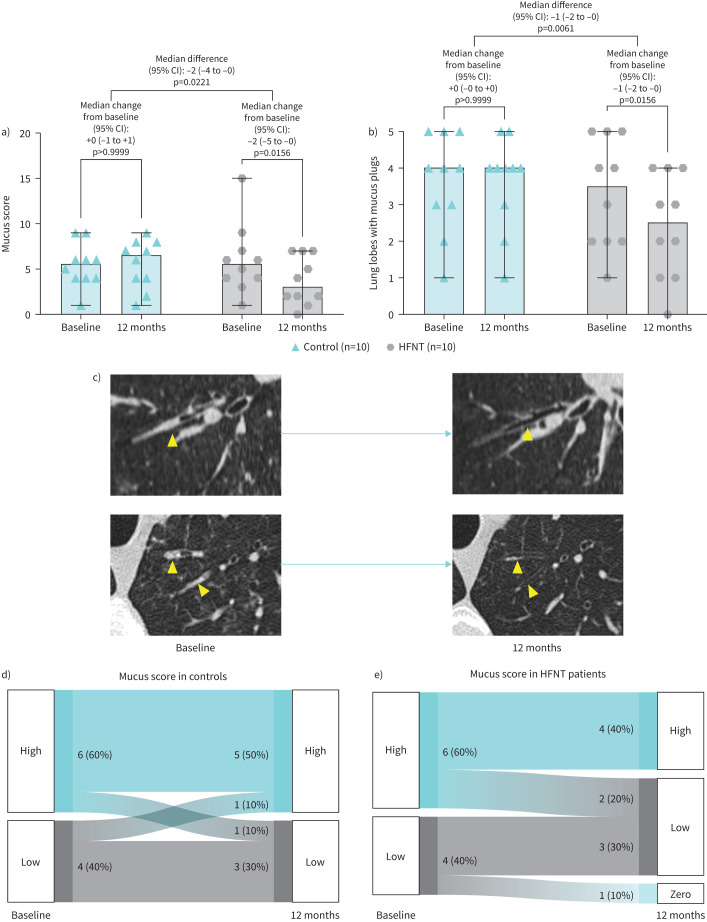
Changes in mucus plugs between baseline and 12 months in patients who received long-term home HFNT compared to controls. a) CT mucus scores, and b) lung lobes with mucus plugs were measured at baseline and after 12 months in both the HFNT and control groups. Every dot represents a single patient. Bars represent median values with interquartile range. c) Representative CT slices acquired at baseline and after 12 months for two different subjects who received HFNT. Yellow arrows identify mucus plugs at baseline that were resolved after 12 months of HFNT. Sankey diagrams illustrate changes in the percentage of d) controls, and e) HFNT patients with high and low mucus scores at baseline and after 12 months. HFNT: high-flow nasal therapy.

Physiologically, airway mucus is composed of 2% mucin and 98% water and their relative proportions are linked to inflammatory responses and mechanisms of disease. Dysregulated mucin expression and increased sputum mucin concentration are tightly associated with muco-obstructive disease [5, 15]. At the same time, proper mucus hydration is essential for its transport and clearance, as even slight water content changes can substantially affect its rheological characteristics. Increased mucus production and viscosity, combined with impaired mucus clearance, can lead to mucus plug formation, resulting in airway occlusion that contributes to air trapping, airflow limitation and obstruction [16]. Additionally, mucus plugs can cause airway epithelial hypoxia, altering ion transport and causing further dehydration. This hampers the oxygen-dependent antimicrobial activities of macrophages and neutrophils, promotes anaerobic microorganism infection and increases the risk of exacerbations [15].

In this *post-hoc* analysis we observed significant improvements in CT mucus score of patients with bronchiectasis treated with long-term HFNT. These preliminary novel findings suggest that long-term HFNT, by restoring mucus hydration and improving mucociliary clearance, disrupts airways luminal mucus plugs that are known to be associated with increased risk of exacerbations and poor health-related quality of life in muco-obstructive diseases [7, 16]. Thus, reduction of exacerbations in patients receiving HFNT may be plausibly linked to dissolution of mucus plugs, and visual CT-scan assessment of mucus plugs could serve as a valuable imaging tool for disease phenotyping, clinical outcomes and treatment response of therapies targeting mucus dysfunction. Limitations of the study are the retrospective design and the small sample size, which limit the external validity and generalisability of our findings. Future research should be focused on assessing the role of HFNT in patients with bronchiectasis as a direct therapeutic measure.
